# Genetic Diversity Studies Based on Morphological Variability, Pathogenicity and Molecular Phylogeny of the *Sclerotinia sclerotiorum* Population From Indian Mustard (*Brassica juncea*)

**DOI:** 10.3389/fmicb.2018.01169

**Published:** 2018-06-05

**Authors:** Pankaj Sharma, Amos Samkumar, Mahesh Rao, Vijay V. Singh, Lakshman Prasad, Dwijesh C. Mishra, Ramcharan Bhattacharya, Navin C. Gupta

**Affiliations:** ^1^Sclerotinia Lab, ICAR, Directorate of Rapeseed and Mustard Research, Bharatpur, India; ^2^Brassica Lab, ICAR, National Research Centre on Plant Biotechnology, New Delhi, India; ^3^ICAR, Indian Agricultural Research Institute, New Delhi, India; ^4^ICAR, Indian Agricultural Statistics Research Institute, New Delhi, India

**Keywords:** morphological, molecular, phylogeny, *Sclerotinia sclerotiorum*, stem rot, *Brassica*, diversity

## Abstract

White mold or stem rot disease are ubiquitously distributed throughout the world and the causal organism of this disease *Sclerotinia sclerotiorum* (Lib.) de Bary, is known to infect over 400 plant species. Sclerotinia stem rot is one of the most devastating fungal diseases and poses a serious threat to the worldwide cultivation of oilseed *Brassica* including India. *S. sclerotiorum* pathogen usually infects the stem but in severe cases leaves and pods also affected at different developmental stages that deteriorate not only the oil quality but also causing the seed and oil yield losses up to 90% depending on the severity of the disease infestation. This study investigated the morphological and molecular characterization of pathogenic *S. sclerotiorum* (Lib) de Bary geographical isolates from oilseed *Brassica* including *Brassica juncea* (Indian mustard). The aim of this study was to compare isolates of *S. sclerotiorum* originated from different agro-climatic conditions and to analyse similarity or differences between them as well as to examine the virulence of this pathogen specifically in *Brassica* for the first time. The collection of *S. sclerotiorum* isolates from symptomatic *Brassica* plants was done and analyzed for morphological features, and molecular characterization. The virulence evaluation test of 65 isolates on four *Brassica* cultivars has shown 5 of them were highly virulent, 46 were virulent and 14 were moderately virulent. Phylogenetic analysis encompassing all the morphological features, SSR polymorphism, and ITS sequencing has shown the existence of high genetic diversity among the isolates that categorized all the isolates in three evolutionary lineages in the derived dendrogram. Further, genetic variability analysis based on sequences variation in ITS region of all the isolates has shown the existence of either insertions or deletions of the nucleotides in the ITS region has led to the interspecies variability and observed the variation were in a clade-specific manner. Together this analysis observed the existence of higher heterogeneity and genetic variability in *S. sclerotiorum* isolates collection and indicates the presence of clonal and sexual progenies of the pathogen in the mustard growing regions of India surveyed in this study. With a higher level of genetic variability and diversity among the *S. sclerotiorum* population needs robust screening approaches to identify the donor parent and utilize them in resistance breeding program for effectively counter the menace of stem rot disease in *Brassica*.

## Introduction

Globally India continues to be at a 3rd position after Canada and China in acreage (19.3%) and after China and Canada in production (11.1%) of rapeseed-mustard. In India, among nine edible oilseed crops, the share of rapeseed-mustard is about one-fourth of total area and one-third of total oil production in the country. During 2015–2016, production (6.82 mt) and productivity (1184 kg/ha) was achieved (Anonymous, 2016). Rapeseed-mustard is the major source of income especially for the marginal and small farmers in rainfed areas which are about 25% of the total cultivated area. In spite of its increase in demand for the year the production of oilseed Brassica remains to stagnate over the year and most of the demands are being met through import from outside the India. The main reason behind productivity stagnation in Indian Brassica is its susceptibility and damages caused to the crop by various insect pests and disease infestation in addition to the other yield-limiting factors. Out of thirty diseases known to infest the Brassica crops in India, stem rot has been found one of the most devastating diseases that heavily damages the crops during the flowering stage of development. The stem rot disease which is caused by fungal pathogens, *Sclerotinia sclerotiorum* (Lib) de Bary, ubiquitously found throughout the world is a polyphagous, soil-borne plant pathogen that infects more than 400 plant species of diverse phylogenetic origin (Boland and Hall, 1994; Saharan and Mehta, 2008; Sharma et al., 2015). In India, during the eighties and nineties, the stem rot (SR) disease in rapeseed-mustard was of a minor importance, because of its seldom appearance over the ground level of the isolated plants after mycelial infection. A widely adopted monocropping practices and cultivation of rapeseed-mustard under irrigated condition has significantly increased the sclerotial population in the soil that has made SR very serious disease of oilseed *Brassica* crops in states including Rajasthan, Haryana, Punjab, Uttar Pradesh, Bihar, Assam, West Bengal and Madhya Pradesh (Sharma et al., 2015). This fungus has been long considered as prototypical necrotrophs as it begins highly pathogenic phase by releasing oxalic acids and cellulolytic enzymes immediately upon host cuticle penetration followed by mycelial proliferation inside the host cell followed by a saprophytic phase that supports the sclerotia formation (Hegedus and Rimmer, 2005). However, the recent studies decipher the fact of evidence for the occurrence of a brief biotrophic phase just within the apoplastic space next after the establishment of the host-pathogen connection and hence based on these it is more appropriately classified as a hemibiotroph (Kabbage et al., 2015). The information related to the genetic diversity of the pathogen and their effective virulence over the target crop is the foremost requirement for taking the breeding program for development of pathogen resistance in the release of the region-specific cultivars. Various diversity analysis tools based on the molecular methods like microsatellite haplotype (Aldrich-Wolfe et al., 2015), SSR (simple sequence repeat or microsatellite-based marker; Meinhardt et al., 2002), AFLP (amplified fragment length polymorphism; Cubeta et al., 1997), and SRAP (sequence-related amplified polymorphism technique; Li et al., 2009) have been used in analyzing the genetic diversity of the pathogen *S. sclerotiorum* from different host species. Very limited variability was observed in ITS (internal transcribed spacer) sequences in *S. sclerotiorum* isolates from various host species (Njambere et al., 2008) and thus a universal barcode markers were developed from the nearly conserved nature of the nuclear ribosomal internal transcribed spacer (ITS) region for imparting the individual identity to the fungus up to genus level (Schoch et al., 2012). Furthermore, MCGs is another diversity analysis method based on the mycelial compatibility grouping (MCG) has been used in establishing the kinship among *S. sclerotiorum* isolates from chickpea (Kull et al., 2004; Li et al., 2008). In addition to it, the diversity based on the morphological appearance of sclerotia, mycelial growth, and ascospores formation have also been reported in analyzing the genetic diversity of *S. sclerotiorum* isolates in previous studies (Li et al., 2008; Sharma et al., 2013). However, polymorphism and genetic diversity of the *S. sclerotiorum* isolates at the morphological and DNA sequence level has not been comprehensively studied so far especially for the isolates from Brassica species of India.

Being a polyphagous nature, *S. sclerotiorum* pathogen is usually infecting not only the majority of the economically important dicotyledonous species but also serve as the major pathogen for several monocotyledonous plant species (Boland and Hall, 1994). The yield loss estimated with the *S. sclerotiorum* infestation has exceeded hundred million dollars annually because of the lack of resistance cultivar of the crop species and also because of lack of the effective management practices (Tok et al., 2016). In general, the management of *S. sclerotiorum* borne disease is not much easy because of its widespread existence, irregular incidence, and the long-term survival by producing huge numbers of sclerotia in the soil. Although several control measures like chemical and cultural methods have been devised and adopted for countering the Sclerotinia stem rot menace (Rousseau et al., 2007) none of them were found fully effective in preventing either the process of disease infection or pathogenesis progression after infection. The extent of genetic diversity of the pathogen and their widespread distribution among host species across the growing regions play an important role in determining and devising the control strategies to efficiently control the diseases in the more effective way. Hence, the availability of the genetic variability information related to the target pathogen is the foremost requirement for designing the management means to counter the disease incidence more effectively. In plant-pathogen interactions, development of new pathogenic races, and the breakdown of host resistance are the limiting factors in resistance deployment against plant diseases. The pathogen's life history characteristics and evolutionary potential are major factors leading to the pathogen overcoming host resistance. Therefore, major efforts should be focused not only in understanding the genetic structure of the fungal populations but also to determine how populations will evolve in response to different control strategies (McDonald and Linde, 2002).

In recent past, India observed the frequent incidence of the *S. sclerotiorum* infestation on cereals and horticultural species that draws the wide attention of the researcher over this fungal pathogen. In pursuance of basic understanding about pathogenicity, diversity and distribution pattern of the *S. sclerotiorum* were extensively studied on isolates collected from various host species like chickpea (Mandal and Dubey, 2012), vegetable crops (Choudhary and Prasad, 2012), cumin (Prasad et al., 2017), carnation (Kumar et al., 2015), oilseed *Brassica* (Sharma et al., 2013), and their identity were established on the basis of morphological features and cultural conditions. However, major variation in the growth characteristics has been reported in the growing collection of *S. sclerotiorum* isolates even as they belong to the same *Sclerotinia* species. Indeed, as projected with diversity analysis *S. sclerotiorum* isolates has been reported of possessing the variation in morpho-physiological, biochemical properties, molecular features and pathogenicity in terms of virulence because of the presence of clonal and sexual progenies together even in the same crop and in same region (Atallah et al., 2004; Sexton and Howlett, 2004; Irani et al., 2011). For establishing the console features of the pathogen especially in *Brassica* growing regions of India, the present study was aimed at determining the genetic diversity within *S. sclerotiorum* population from various *Brassica* species based on morphological characteristics, genotyping with simple sequence repeats (SSR) markers and molecular phylogeny by ITS sequence analysis.

## Materials and methods

### *Sclerotinia sclerotiorum* isolates

*Brassica* growing areas in 10 states of India (Rajasthan, Haryana, Punjab, Delhi, U.P., Bihar, Uttarakhand, Himachal Pradesh, Jammu & Kashmir, and Jharkhand) were surveyed and stem rot disease infected plants were collected from 65 different locations (Table 1). The sclerotia obtained from the stem rot infested Indian mustard plant samples were first washed with sterile water than surface sterilized with 70% ethanol for 2 min and again washed two times with sterile water. The drained sclerotia over pre-sterilized filter papers were placed on nutrient media (Potato Dextrose Agar; PDA) plates supplemented with 50 μg/ml tetracycline antibiotics to prevent the growth of bacterial contamination. The samples were wrapped in a brown envelope and kept for incubation at 20° ± 2°C in dark for 4–5 days. After the development of the white fluffy mass of mycelial growth of *S. sclerotiorum*, the mycelial plaques were used to sub-culture the isolates on PDA slants and pure culture of them was stored at 4°C for future use. Morphological identification of the isolates was based on cultural characteristics of the *S. sclerotiorum* and morphology of the mycelial mat and sclerotia formation traits.

**Table 1:** *Sclerotinia sclerotiorum* (Lib) de Bary population used for studying the virulence and genetic diversity within isolates based on morphological features, SSR profiling, and ITS sequence analysis.

**Strain No**.	**Source (Host)**	**Geographic origin**	**Latitude and Longitude**	**Province**	**Disease severity observed in** ***Brassica*** **cultivars (Lesion length cm)**				
					***B. juncea* (NRCDR-2)**	***B. rapa var* Toria (Uttara)**	***B. rapa var yellow sarson* (NRCYS 5-2)**	***B. rapa var brown sarson***	**Mean**
ESR 1	*B. juncea*	DRMR, Bharatpur	27°15′N; 77°30′E	Rajasthan	14.0	18.7	21.0	17.0	17.7
ESR 2	*B. juncea*	Oraiya	26°46′N; 79°51′ E	UP	13.7	13.0	18.7	13.0	14.6
ESR 3	*B. rapa*	Imlia, Varansai	26°4′N; 77°9′E	UP	15.7	13.7	20.0	14.7	16.0
ESR 4	*B. juncea*	CSA, Kanpur	26°28′N; 80° 24′E	UP	6.5	8.0	8.0	8.3	7.7
ESR 5	*B.rapa*	Allahbad	25°28′N; 81° 54′E	UP	12.5	16.0	17.3	12.0	14.5
ESR 6	*B. juncea*	BHU, Varanasi	25°26′N; 82°9′E	UP	12.0	10.0	18.7	16.7	14.4
ESR 7	*B. juncea*	Akbarpur, Kanpur	26°26′N; 52° 33′E	UP	14.7	13.0	23.0	12.7	15.9
ESR 8	*B. juncea*	Etawah	26°47′N; 79° 02′E	UP	5.7	12.7	16.7	10.0	11.3
ESR 9	*B. juncea*	Firozabad	27°09′N; 78° 24′E	UP	14.0	18.3	25.0	14.3	17.9
ESR 10	*B. juncea*	Khaga	25°47′N; 81°07′E	UP	9.7	8.7	13.7	8.7	10.2
ESR 11	*B. juncea*	Kokhraj	26°55′N; 80°59′E	UP	5.0	4.7	6.3	4.7	5.2
ESR 12	*B. juncea*	Koshambi	25°48′N; 81°66′E	UP	16.7	14.0	22.7	15.0	17.1
ESR 13	*B. rapa*	Meghipur	24°94′N; 85.69 E	Bihar	11.7	13.7	19.3	14.3	14.8
ESR 14	*B. juncea*	Morena	26°49′N; 77°76′E	MP	8.0	6.3	8.0	4.7	6.8
ESR 15	*B. juncea*	Handia	25°38′N; 82°18′E	UP	19.0	24.3	25.0	21.0	22.3
ESR 16	*B. juncea*	Bawal	28°08′N; 76°58′E	Haryana	14.0	13.3	18.0	19.0	16.1
ESR 17	*B. juncea*	Maluka	30°21′N; 74°96′E	Punjab	17.7	17.0	24.0	20.7	19.9
ESR 18	*B. juncea*	Bhatinda	30°11′N; 75°00′E	Punjab	27.7	22.7	32.7	20.0	25.8
ESR 19	*B. juncea*	Moga	30°48′N; 75°10′E	Punjab	16.0	14.0	21.7	11.3	15.8
ESR 20	*B. juncea*	PAU, Ludhiana	30°55′N; 75°54′E	Punjab	15.0	13.3	25.7	14.0	17.0
ESR 21	*B. juncea*	Faridkot	30°67′N; 74°73′E	Punjab	11.3	15.0	19.3	10.0	13.9
ESR 22	*B. juncea*	Mahalkalan, Sangrur	30°75′N; 76°78′E	Punjab	13.7	16.3	20.7	17.3	17.0
ESR 23	*B. juncea*	Rajoana, Ludhiana	30°72′N; 75°6′E	Punjab	8.0	15.0	22.7	12.7	14.6
ESR 24	*B. juncea*	Ranchi	23°23′N; 85° 23′E	Jharkhand	4.3	4.0	9.0	4.0	5.3
ESR 25	*B. juncea*	Mathura	27°28′N; 77°41′E	UP	19.7	26.0	28.7	24.7	24.8
ESR 26	*B. juncea*	CCSHAU Hisar	29°14′N; 75°72′E	Haryana	13.0	16.0	20.0	17.0	16.5
ESR 27	*B. juncea*	Sirsa	29°53′N; 75°01′E	Haryana	14.0	12.3	20.0	14.0	15.1
ESR 28	*B. juncea*	Bassi, Jaipur	26°91′N; 75°78′E	Rajasthan	5.3	4.0	9.0	4.3	5.7
ESR 29	*B. juncea*	Shahganj, Janunpur	24°70′N; 82°95′E	UP	6.0	11.7	18.7	10.7	11.8
ESR 30	*B. juncea*	Jammu	31°14′29″N; 77°2′12″E	J & K	14.3	18.0	25.3	18.7	19.1
ESR 31	*B. juncea*	Bansur, Alwar	27°71′N; 76°28′E	Rajasthan	15.3	15.0	21.7	15.3	16.8
ESR 32	*B. juncea*	Badodamev, Alwar	27°34′N; 76° 38′E	Rajasthan	13.7	12.3	23.7	9.7	14.9
ESR 33	*B. juncea*	DU, SC	28°36′N; 77°22′E	Delhi	11.7	16.0	18.0	14.3	15.0
ESR 34	*B. juncea*	Pantnagar	29°02′N; 79°48′E	Uttarakhand	11.0	15.0	15.7	10.0	12.9
ESR 35	*B. juncea*	Kherli	27°20′N; 77°03′E	Rajasthan	23.7	23.3	31.3	22.0	25.1
ESR 36	*B. juncea*	Amoli	27°09′N; 77°08′E	Rajasthan	49.0	46.3	55.0	42.3	48.2
ESR 37	*B. juncea*	Dausa	26°89′N; 76°33′E	Rajasthan	11.5	11.3	19.0	12.0	13.5
ESR 38	*B. juncea*	Nadbai	27°21′N; 77°20′E	Rajasthan	4.3	14.7	14.7	13.3	11.8
ESR 39	*B. juncea*	Ludhawai	27°21′N; 77°2′E	Rajasthan	11.3	18.7	21.3	14.0	16.3
ESR 40	*B. juncea*	Sangaria,SGN	29°79′N; 74°46′E	Rajasthan	5.7	12.7	16.7	10.0	11.3
ESR 41	*B. juncea*	Sri Ganga nagar	29°90′N; 73°87′E	Rajasthan	8.0	12.7	16.7	10.0	11.9
ESR 42	*B. juncea*	Kumher	27°34′N; 77°37′E	Rajasthan	5.3	7.0	13.0	10.0	8.8
ESR 43	*B. juncea*	Gajsinghpur	29°65′N; 73°43′E	Rajasthan	8.3	16.0	17.7	12.0	13.5
ESR 44	*B. juncea*	Halena	27°10′N; 77°15′E	Rajasthan	8.0	11.7	15.3	10.0	11.3
ESR 45	*B. juncea*	Bajoli	26°86′N; 74°4′E	Rajasthan	9.7	18.3	15.3	13.3	14.2
ESR 46	*B. juncea*	Mahua	27°05′N; 76°92′E	Rajasthan	7.3	12.3	17.0	15.3	13.0
ESR 47	*B. juncea*	Kiratpura, Jaipur	31°18′N; 76°.56′E	Punjab	4.7	11.0	11.3	12.7	9.9
ESR 48	*B. juncea*	Pathredi	27°59′N; 76°09′E	Rajasthan	8.7	10.7	12.0	11.0	10.6
ESR 49	*B. juncea*	Bhadubha	26°89′N; 75°81′E	Rajasthan	4.3	13.7	14.0	14.0	11.5
ESR 50	*B. juncea*	Tulsipura	27°57′N; 82°45′E	UP	2.0	14.0	12.0	11.3	9.8
ESR 51	*B. juncea*	Chhonkarwara	27°10′N; 77°04′E	Rajasthan	9.7	14.3	19.7	14.3	14.5
ESR 52	*B. juncea*	Kaimasi	27°08′N; 77°38′E	Rajasthan	5.0	14.3	16.0	10.7	11.5
ESR 53	*B. juncea*	Habibpur	25°21′N; 86°98′E	UP	1.7	12.3	12.3	12.3	9.7
ESR 54	*B. juncea*	Baansi	24°32′N; 74°38′E	Rajasthan	2.3	6.3	10.0	10.7	7.3
ESR 55	*B. juncea*	Jhalatala	27°08′N; 77°01′E	Rajasthan	1.7	16.0	14.7	11.7	11.0
ESR 56	*B. juncea*	Gundwa	27°24′N; 77°46′E	Rajasthan	1.3	2.0	7.3	4.3	3.7
ESR 57	*B. juncea*	Deeg	27°34′N; 77°37′E	Rajasthan	4.0	1.3	4.7	2.7	3.2
ESR 58	*B. juncea*	Sewar	27°18′N; 77°44′E	Rajasthan	8.0	12.0	13.0	8.7	10.4
ESR 59	*B. juncea*	Hantra	27°12′N; 77°24′E	Rajasthan	2.7	4.3	6.0	4.0	4.3
ESR 60	*B. juncea*	Kanma	27°08′N; 77°1′E	Rajasthan	2.0	10.0	13.0	9.0	8.5
ESR 61	*B. juncea*	Baansi	24°32′N; 74°38′E	Rajasthan	12.7	11.3	18.7	11.7	13.6
ESR 62	*B. juncea*	Dehra mod	27°21′N; 77°49′E	Rajasthan	2.0	2.0	6.0	4.7	3.7
ESR 63	*B. juncea*	Aroda	23°75′N; 72°89′E	Rajasthan	15.0	15.0	25.7	15.0	17.7
ESR 64	*B. juncea*	Bhandor	27°26′N; 77°45′E	Haryana	6.0	8.7	12.3	11.0	9.5
ESR 65	*B. juncea*	Basua	25°02′N; 87°32′E	Rajasthan	16.0	18.0	26.0	18.0	19.5

The severity of disease assessed on Brassica cultivars was measured in lesion length (cm). Disease index for the strains listed, accessed on length of the lesion developed on stem (in cm). Lesion size 0.0 cm: Non-virulent; 0.1–3.0 cm: less virulent; 3.1–10.0: Moderately virulent; 10.1–20.0 cm: virulent; >20.0 cm: highly virulent. NRCDR-02 stands for National Research Centre Disease Resistance-02, YS stands for yellow sarson

### Morphological characterization of *S. sclerotiorum* isolates

Freshly grown 3–4 days old cultures of *Sclerotinia* isolates were used for analyzing the morphological features like the radial spread of mycelial growth (mm) at 72 and 96 h after inoculation. Whereas, the number of sclerotia produced from each inoculum per Petri plates (90 mm), length and diameter of the sclerotia (mm) and weight of individual sclerotia (mg) was measured 10–15 days after inoculation.

### Pathogenicity of isolates

An experiment to study pathogenicity and pathogenic variability among 65 geographical isolates of *S. sclerotiorum* were conducted during 2016–2017 post-rainy season at ICAR-Directorate of Rapeseed-Mustard Research, Bharatpur, India. Seven different oilseed *Brassica species* were selected for this study. The crop was destroyed after the experiment by cutting and collecting the debris followed by autoclaving before disposing of them.

### Inoculation

The inoculum was mass multiplied in the laboratory on autoclaved sorghum grains in glass jars and incorporated into the soil prior to sowing. Further, the plants were inoculated at 60 days after sowing on the stem with the pathogen growing on agar blocks. Stem inoculation procedure was followed as described by Buchwaldt et al. (2003). A single 5 mm mycelial bit cut from *S. sclerotiorum* colony of 4–5 days old culture growing on potato dextrose agar was used to inoculate each plant. The mycelial bit along with cotton swab soaked in sterilized distilled water was placed on a small piece of parafilm (5–7 cm). Mycelial bit touching the stem at 15 cm height was then secured by wrapping the parafilm strip around the stem. Wet cotton swab maintained high humidity during the infection period (Sharma et al., 2013).

### Disease

Disease incidence was assessed by recording the size of stem lesion length (cm) and disease severity (%) 15–21 days after inoculation. This has been shown to be an ideal time to demonstrate the host response to the pathogen (Li et al., 2007). The disease parameters recorded were statistically verified by data analysis using Duncan's test (*P* < 0.05) and analysis of variance (ANOVA).

### DNA extraction

*Sclerotinia sclerotiorum* isolates were grown on PDA medium at 20° ± 2°C in BOD incubator for 5 days. Mycelial mat was harvested by scraping with a sterile spatula and ~2 g of the mycelial mat was used for DNA extraction using modified CTAB method. The fine powder of the grounded mycelial mat in liquid nitrogen was transferred in preheated 10 ml DNA extraction buffer (1% Cetyltrimethylammonium bromide; 250 mM NaCl; 100 mM Tris-HCl, pH 8.0; 100 mM EDTA, pH 8.0; along with 4 mM Spermidine) and incubated at 65°C with intermittent mixing at regular interval for 60 min. Denatured proteins were removed by extracting once with an equal volume of Tris-saturated phenol: chloroform: isoamyl alcohol (25:24:1, v/v/v), followed by repeated extractions with an equal volume of Tris-saturated chloroform: isoamyl alcohol (24:1, v/v). The aqueous phase obtained after centrifugation was added with 0.6 volume of chilled isopropanol and stored overnight at −20°C for DNA precipitation. DNA was pelleted, dried and resuspended in 500 μl of Tris–EDTA buffer [10 mM Tris-HCl, pH 7.5; 1 mM EDTA (ethylenediaminetetraacetic acid)]. The purity and concentration of the DNA were determined through NanoDrop (Thermo Scientific, USA) and stored in aliquots at −20°C.

### Species-specific PCR assay

*Sclerotinia*-specific primers as described by Freeman et al. (2002) (Table 2) were used to carry out PCR amplification in all the isolates. The PCR reaction of 15 μL contained 1.5 μL of 10x PCR buffer with 15 mM MgCl_2_, 50 μM dNTPs, 10 μM forward and reverse primers, 1 U Taq DNA polymerase (Bangalore Genei, India) and 50 ng of template DNA was performed in a thermal cycler (Biometra, ILS, USA) and the program made with initial denaturation at 95°C for 4 min followed by 35 cycles of denaturation at 94°C for 45 s, annealing at 57°C for 45 s and extension at 72°C for 1 min, and a final extension at 72°C for 10 min. The PCR amplicons were resolved on 1% agarose gel along with 1 kb standard DNA ladder and visualized in a UV transilluminator. Gels were photographed using Alpha image (Alpha innotech, USA) Gel Doc system.

**Table 2:** Details of the primers used for identification of *Sclerotinia* species, rDNA conserved sequence, and ITS analysis.

**Primer**	**Primer Sequence (5^′^-3^′^)**	**Amplification size**	**Reference**
SSFWD	GCTGCTCTTCGGGGCCTTGTATGC	278 bp	Freeman et al., 2002
SSREV	TGACATGGACTCAATACCAAGCTG		
ITS4	TCCTCCGCTTATTGATATGC	600 bp	White et al., 1990
ITS5	GGAAGTAAAAGTCGTAACAAGG		

### Genetic diversity

#### Simple sequence repeat (SSR) marker analysis

The pathogenicity tested 65 *S. sclerotiorum* isolates were subjected to SSR fingerprinting. A total of 25 SSR primers (Sirjusingh and Kohn, 2001; Table 3) were synthesized from IDT, Agrigenome, Bengaluru, India and used for amplification of microsatellite loci. PCR reactions were performed using a BioRad C1000 thermocycler (BioRad, USA). The PCR reaction was set up in a 25 μL reaction volume with 2.5 μL of 10x Taq buffer (with 15 mM MgCl_2_), 0.25 mM dNTPs (10 mM dNTPs mix), 10 nM of forward and reverse primers, 1 U of Taq polymerase and 200 ng template DNA. The reproducibility of the amplification was confirmed by repeated PCR with a similar set of ingredients and condition. For each experiment, negative controls were taken with sterile water in place of the template. The thermal cycler program consisted of an initial denaturation for 4 min at 95°C, followed by 35 cycles of denaturation at 94°C for 45 s, optimized annealing temperature for 45 s, and extension at 72°C for 1 min with a final extension of 72°C for 10 min. The PCR amplicons were resolved on 3.5% agarose gel in 1x TAE buffer by electrophoresis at 60 V cm^−1^.

**Table 3:** Optimized annealing temperature (Tm), Polymorphism information content (PIC), product size and a total number of bands observed for the SSR markers used to check the presence genetic diversity among *S. sclerotiorum* isolates.

**Primer ID**	**Primer sequence (5^′^-3^′^)**	Locus^*^	**Repeat motif ^#^**	**Tm (Â°C)**	**PIC**	**Amplicon size range**	**Allele frequency**
SS 1A	CCGAGCATAATATACATCC	41-1	(TA)5 and (CA)10	50	0.663	494–504	1
	AAGGTTATATTTCCCTCGC						
SS 2B	GTAACACCGAAATGACGGC	5-2	(GT)8	55	0.467	318–325	1
	GATCACATGTTTATCCCTGGC						
SS 3C	GGGGGAAAGGGATAAAGAAAAG	6-2	(TTTTTC)2(TTTTTG)2(TTTTTC)	55	0.564	479–484	1
	CAGACAGGATTATAAGCTTGGTCAC						
SS 4D	TTTGCGTATTATGGTGGGC	7-2	(GA)14	50	0.345	160–172	1
	ATGGCGCAACTCTCAATAGG						
SS 5E	GCCGATATGGACAATGTACACC	9-2	(CA)9(CT)9	50	0.243	358–382	1
	TCTTCGCAGCTCGACAAGG						
SS 6F	CTTTCCTTTCGTTTGAGGG	11-2	(GA)6GG(GA)6(GGGA)2	47	0.806	276–284	1
	GGCAGGTAATGTTGCTTGG						
SS 7G	CGATAATTTCCCCTCACTTGC	12-2	(CA)9	55	0.260	215–225	1
	GGAAGTCCTGATATCGTTGAGG						
SS 8H	TCTACCCAAGCTTCAGTATTCC	13-2	(GTGGT)6	50	0.391	284–304	1
	GAACTGGTTAATTGTCTCGG						
SS 9I	CAGACGAATGAGAAGCGAAC	5-3	[(GT)2GAT]3(GT)14GAT(GT)5[GAT(GT)4]3(GAT)3	55	0.343	245–320	2
	TTCAAAACAACGCTCCTGG						
SS 10J	CCTGATATCGTTGAGGTCG	7-3	GT10	55	0.207	202–212	1
	ATTTCCCCTCACTTGCTCC						
SS 11K	CACTCGCTTCTCCATCTCC	8-3	CA12	55	0.190	251–271	1
	GCTTGATTAGTTGGTTGGCA						
SS 12L	TCATAGTGAGTGCATGATGCC	17-3	(TTA)9	55	0.603	345–390	4
	CAGGGATGACTTTGGAATGG						
SS 13M	GACGCCTTGAAGTTCTCTTCC	20-4	(GT)7GG(GT)5	50	0.030	268–278	1
	CGAACAAGTATCCTCGTACCG						
SS 14N	CTTCTAGAGGACTTGGTTTTGG	23-4	(TG)10	60	0.360	384–388	4
	CGGAGGTCATTGGGAGTACG						
SS 15O	GAATCTCTGTCCCACCATCG	36-4	CA6(CGCA)2CAT2	50	0.097	415–429	1
	AGCCCATGTTTGGTTGTACG						
SS 16P	GGTCTCATACAGTCTACACACA	42-4	GA9	60	0.360	410–414	1
	CTCTAGAGGATCTGCTGACA						
SS 17Q	CCCTACAATATCCCATGGAGTC	50-4	CA7(TACA)2	50	0.243	419–527	2
	CCTCGTCTATCCGTCCATC						
SS 18R	GTTTTCGGTTGTGTGCTGG	55-4	TACA10	50	0.153	173–221	1
	GCTCGTTCAAGCTCAGCAAG						
SS 19S	TCGCCTCAGAAGAATGTGC	92-4	(CT)12	50	0.153	374–378	1
	AGCGGGTTACAAGGAGATGG						
SS 20T	CTCATTTCATCCCATCTCTCC	99-4	(GTAA)2(GCAA)(GTAA)3	50	0.243	402–422	1
	AATTCAAGCCTTCCTCAGCC						
SS 21 U	TGCATCTCGATGCTTGAATC	106-4	(CATA)25	55	0.391	491–571	1
	CCTGCAGGGAGAAACATCAC						
SS 22V	ATCCCTAACATCCCTAACGC	110-4	(TATG)9	50	0.294	362–378	1
	GGAGAATTGAAGAATTGAATGC						
SS 23W	GCTCCTGTATACCATGTCTTG	114-4	(AGAT)14(AAGC)4	50	0.294	351–391	1
	GGACTTTCGGACATGATGAT						
SS 24X	TCAAGTACAGCATTTGC	117-4	(TAC)6C(TAC)3	55	0.564	376–388	1
	TTCCAGTCATTACCTACTAC						
SS 25Y	GTAACAAGAGACCAAAATTCGG	119-4	(GTAT)6 and (TACA)5	50	0.135	369–391	1
	TGAACGAGCTGTCATTCCC						

* Locus names;

# *Repeat motifs as mentioned by Sirjusingh and Kohn (2001)*.

### SSR profiling and data analysis:

The standard binomial matrices were followed in SSR profiling and the presence and absence of the allele at a particular locus was scored as 1 and 0, respectively. Though Jaccard's coefficient pairwise distance were calculated and the resultant distance matrices were further used in NTSYSpc2 software version 2.0 (Rohlf, 2000) for clustering the isolates under investigation based on the Unweighted Pair Group method using arithmetic means (UPGMA) method. Allele frequency and Polymorphism information content (PIC) value for each marker was also calculated based on the formula: *Hn* = 1–Σ*pi*2, where *pi* is the allele frequency of the *i*th allele (Nei, 1973).

### Internal transcribed Spacer (ITS) amplification

The universal primers pair ITS4 and ITS5 (White et al., 1990; Table 2) were used for amplifying the ITS region of the rDNA. The PCR was carried out in a total volume of 50 μL with 1xPCR buffer (with 15 mM MgCl_2_), 250 μM each dNTPs, 1 μL of each primer (10 nmol), 1.5 U Taq DNA polymerase, and 200 ng of genomic DNA. The thermal cycler program consisted of an initial denaturation at 95°C for 4 min followed by cycling conditions included denaturation at 94°C for 45 s, annealing at 49°C for 45 s, and elongation at 72°C for 1 min (35 cycles), followed by a final extension at 72°C for 10 min. All the PCR reactions were set up along with a negative control (without template DNA). The amplification product was analyzed on 1% agarose gels by running a standard DNA molecular weight 1 kb marker parallel to it.

### rDNA sequence analysis

The PCR amplified internal transcribed (ITS) rDNA region from each of the isolates was purified using Favorgen PCR purification kit (FAVORGEN Biotech Corp, Taiwan), as per the manufacturer's instructions. The purified ITS amplicons were sequenced by Agrigenome, Bengaluru, India and then the sequences were analyzed in GenBank (http://www.ncbi.nlm.nih.gov/) by using the Mega BLAST sequence analysis tool. The full length ITS sequences were used as queries for establishing kinship with the published sequences and search result with maximum homology and highest score were marked for further analysis. All the ITS sequences obtained from the isolates used in the present study were submitted to GenBank. ClustalW program of the Bioedit sequence alignment editor (Thompson et al., 1994; Hall, 1999) was used to align all the obtained sequences. The resulting multiple-alignment file was used for phylogenetic analyses which were performed using Molecular Evolutionary Genetics Analysis (MEGA 7.0) with Neighbour-Joining method (Kumar et al., 2016).

## Results and discussion

### Morphological identification

**Figure 1:**
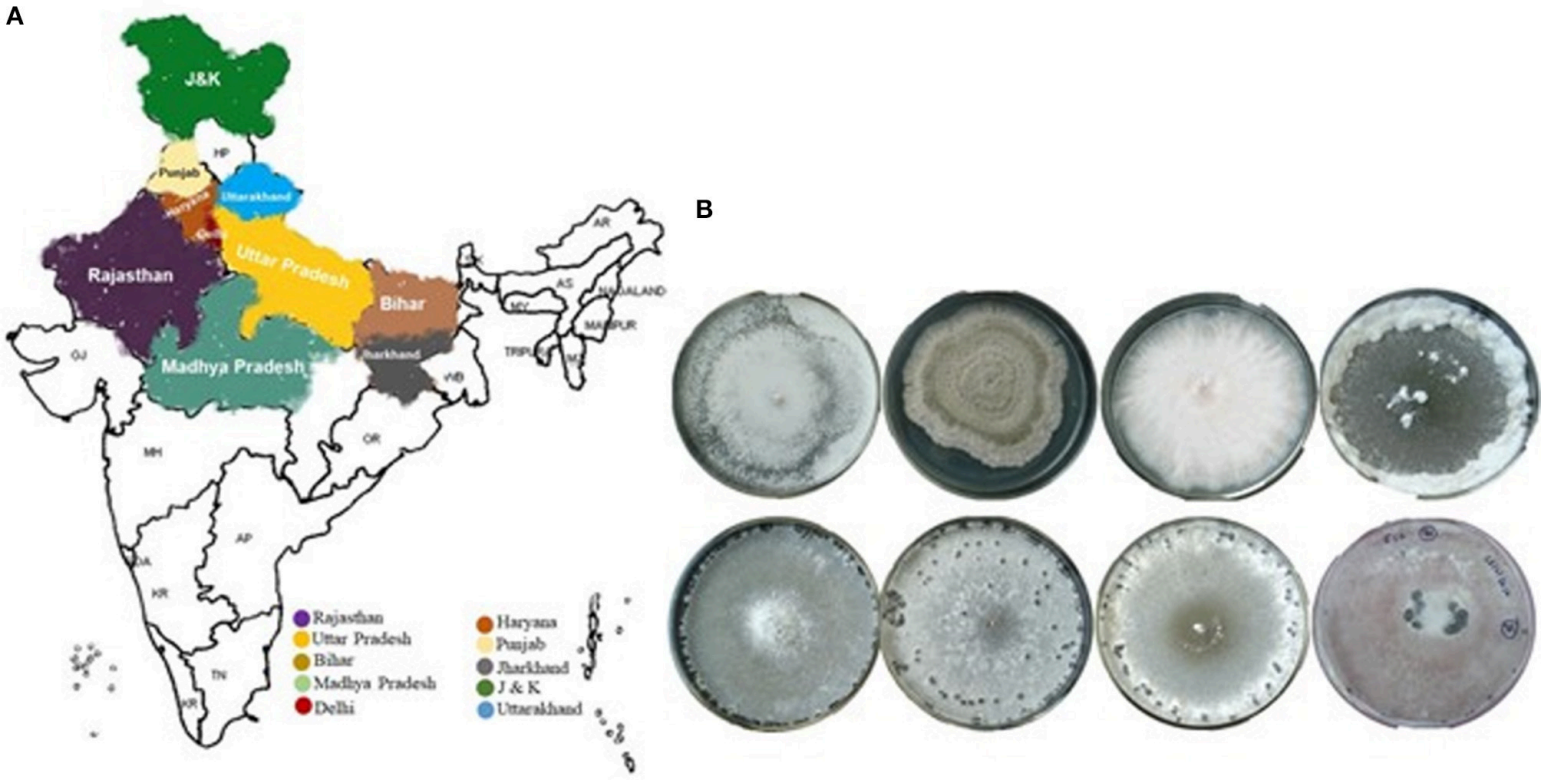
Geographic distribution map of isolates of *Sclerotinia sclerotiorum* from the 10 different states of India. There were 65 isolates collected from *Brassica* field of these states **(A)**, and representative images of the *S*. *sclerotiorum* isolates with morphological variabilities **(B)**.

All the 65 *S. sclerotiorum* isolates collected from the major *Brassica* growing regions of India were observed exhibited morphological characteristics specific to *Sclerotinia sclerotiorum*. The mycelia of *S. sclerotiorum* isolates produced abundantly and appeared white to off-white, fluffy, delicate and generally with a brownish to blackish tinge. Sclerotia produced by the fungus were often dark brown to black and appeared concentrically in some case and at the periphery in others. The sclerotia were formed in abundant at the terminal stage of the growth and vary in its shape and size viz. oval-ellipsoid, straight to curved, 1.8–2.4 × 2.9–6.2 mm in size (Table 4). Usually, the sclerotia were produced solitary in the chain of ring-shaped either at the periphery or in the center and occasionally also formed in pairs randomly. Substantial variability in culture and morphology were exhibited by the *S*. *sclerotiorum* isolates during their growth in a controlled environment (Figure 1).

**Table 4:** Variability in mycelial growth and sclerotia formation (on PDA) in a different geographical isolate of *Sclerotinia sclerotiorum*.

**Isolate**	**Mycelium**				**Sclerotia**						
	**Growth on PDA (mm)**		**Color**	**Texture**	**Scl. Ini. (Days)**	**Days of formation**	**Scl./plate**	**Color**	**Pattern**	**Dia. (mm)**	**Length (mm)**
	**72 h**	**96 hr**									
SR-1	85	90	Whitish	Scattered	4	5	40	Black	Peripheral (Spread)	1.9	3.6
SR-2	90	83	Off White	Scattered	5	5	36	Black	Peripheral	1.9	4.9
SR-3	84	90	Whitish	Scattered	4	5	33	Black	Peripheral	2.0	4.3
SR-4	34	84	Whitish	Scattered	4	6	49	Black	Peripheral (Spread)	1.8	4.2
SR-5	90	90	Whitish	Fluffy	4	5	33	Black	Peripheral	2.2	4.7
SR-6	87	90	Off White	Smooth	4	5	35	Black	Peripheral	2.3	4.1
SR-7	87	90	Dirty White	Scattered	4	5	44	Black	Peripheral	2.0	4.4
SR-8	64	90	Off White	Smooth	5	6	39	Black	Peripheral	2.2	3.5
SR-9	87	90	Whitish	Smooth	4	5	40	Black	Peripheral	1.8	4.6
SR-10	44	61	Whitish	Scattered	5	7	33	Black	Peripheral (Spread)	2.2	2.9
SR-11	80	90	Whitish	Scattered	4	5	35	Black	Peripheral	2.0	4.0
SR-12	87	90	Off White	Scattered	4	5	39	Black	Peripheral (Spread)	2.0	4.2
SR-13	90	90	Whitish	Scattered	4	5	41	Black	Peripheral	1.8	3.5
SR-14	67	90	Off White	Smooth	5	6	38	Black	Peripheral	2.1	6.2
SR-15	87	90	Whitish	Smooth	4	5	40	Black	Peripheral (dou. ring)	2.0	3.4
SR-16	64	80	Whitish	Scattered	5	5	38	Black	Peripheral (dou. ring)	2.1	3.5
SR-17	90	90	Off White	Scattered	4	5	48	Black	Peripheral	2.1	3.1
SR-18	80	90	Whitish	Smooth	4	5	38	Black	Peripheral	1.9	4.9
SR-19	90	90	Whitish	Scattered(fluffy)	4	5	30	Black	Peripheral (Spread)	2.1	2.9
SR-20	90	90	Whitish	Smooth	4	5	62	Black	Peripheral	2.0	3.7
SR-21	90	90	Off White	Scattered	4	5	37	Black	Peripheral	2.1	3.7
SR-22	87	90	Whitish	Smooth	4	5	45	Black	Peripheral (Spread)	2.2	4.4
SR-23	80	90	Whitish	Smooth	4	5	55	Black	Peripheral	2.1	4.5
SR-24	11	34	Whitish	Smooth	6	7	34	Black	Peripheral	2.0	5.9
SR-25	90	90	Whitish	Smooth	4	5	39	Black	Peripheral	2.3	6.3
SR-26	64	84	Dirty White	Scattered(fluffy)	5	5	53	Black	Peripheral (dou. ring)	2.4	4.7
SR-27	84	90	Whitish	Scattered	4	5	37	Black	Peripheral	2.4	5.8
SR-28	67	83	Whitish	Smooth	4	5	39	Black	Peripheral	2.1	4.8
SR-29	70	90	Whitish	Smooth	5	5	32	Black	Peripheral	2.0	6.2
SR-30	47	64	Dirty White	Fluffy	5	5	27	Black	Peripheral	2.1	5.7
SR-31	90	90	Off White	Scattered	4	5	40	Black	Peripheral	2.3	4.6
SR-32	87	90	Dirty White	Smooth	4	5	42	Black	Peripheral	2.1	3.5
SR-33	57	87	Whitish	Fluffy(Scattered)	5	6	44	Black	Peripheral	2.1	4.5
SR-34	73	90	Whitish	Fluffy	5	6	42	Black	Peripheral	2.0	4.6
SR-35	90	90	Dirty White	Smooth	4	5	36	Black	Peripheral	2.3	4.8
SR-36	87	90	Whitish	Scattered	4	5	34	Black	Peripheral (dou. ring)	2.1	4.7
SR-37	90	90	Dirty White	Scattered	4	5	42	Black	Peripheral (Spread)	2.0	4.4
SR-38	70	90	Whitish	Smooth	4	5	50	Black	Peripheral (Spread)	2.2	3.5
SR-39	57	90	Whitish	Scattered	4	5	55	Black	Peripheral (Spread)	2.2	3.7
SR-40	60	90	Whitish	Scattered	5	5	34	Black	Peripheral	2.2	5.3
SR-41	34	87	Whitish	Scattered	5	5	47	Black	Spread (Scatter)	2.2	3.7
SR-42	77	90	Off White	Smooth	5	5	38	Black	Peripheral	2.3	5.4
SR-43	71	90	Whitish	Scattered	5	6	47	Black	Peripheral (dou. ring)	2.1	4.5
SR-44	54	90	Whitish	Fluffy	4	5	32	Black	Peripheral	2.4	3.5
SR-45	70	90	Whitish	Smooth	4	5	47	Black	Peripheral	2.3	4.7
SR-46	60	90	Whitish	Scattered	4	5	52	Black	Peripheral (dou. ring)	2.2	3.1
SR-47	34	87	Whitish	Scattered	5	6	44	Black	Peripheral (dou. ring)	2.0	3.7
SR-48	70	90	Off White	Smooth	4	5	48	Black	Peripheral	2.3	3.5
SR-49	20	77	Whitish	Smooth	5	6	49	Black	Peripheral (dou. ring)	2.4	4.2
SR-50	64	90	Whitish	Fluffy	5	6	25	Black	Peripheral (Spread)	2.0	3.1
SR-51	47	90	Whitish	Scattered	5	5	51	Black	Peripheral (Spread)	2.1	4.5
SR-52	24	77	Whitish	Smooth	5	6	42	Black	Peripheral	2.1	4.9
SR-53	80	90	Whitish	Scattered	4	5	40	Black	Peripheral (dou. ring)	2.2	3.1
SR-54	54	90	Whitish	Scattered	4	5	38	Black	Spread	2.3	3.9
SR-55	60	90	Dirty White	Smooth	5	6	40	Black	Peripheral	2.4	4.3
SR-56	70	90	Whitish	Fluffy	4	5	40	Black	Peripheral (dou. ring)	2.2	4.0
SR-57	67	84	Whitish	Scattered	5	5	36	Black	Peripheral	2.0	3.3
SR-58	77	90	Whitish	Scattered	4	5	59	Black	Peripheral (dou. ring)	2.2	4.5
SR-59	57	90	Whitish	Smooth	4	5	46	Black	Peripheral	2.1	5.3
SR-60	47	90	Whitish	Scattered	5	6	47	Black	Peripheral (dou. ring)	2.1	3.5
SR-61	30	84	Whitish	Fluffy	5	6	50	Black	Peripheral	2.2	3.9
SR-62	50	67	Dirty White	Smooth	4	5	35	Black	Peripheral	2.1	6.3
SR-63	57	90	Whitish	Smooth	4	5	45	Black	Peripheral	2.2	4.7
SR-64	60	90	Whitish	Scattered	4	5	49	Black	Peripheral (Spread)	2.2	4.7
SR-65	40	87	Whitish	Fluffy	4	5	56	Black	Peripheral	2.5	6.6
SE	9.43	5.36			0.24	0.25	5.26			0.14	0.88
CD at 5%	6.50	5.11			0.33	0.33	4.97			0.18	1.17
CV	3.05	0.76			0.03	0.02	1.53			0.02	0.41

*Scl. Ini, Sclerotia initiation; Dia, Diameter; dou. Ring, double ring*.

### Species-specific PCR assay

For ascertaining the originality, the *S. sclerotiorum* isolates was tested by using the species-specific PCR assay. For this, the species-specific primers designed and verified by Freeman et al. (2002) were used and a single 278 bp DNA fragment amplified by PCR was specific to *S. sclerotiorum*, which confirms the species-specific identity of the isolates (Figure S10).

### Morphological variability

The growth rates of mycelia in all the 65 isolates of *S. sclerotiorum* were varied irrespective of their collection from the similar host species and they were prominently distinct in terms of the morphological characteristics. Depending on the growth characteristics of mycelium after 72 h of incubation the isolates were categorized into 3 groups: (i) Fast growing (ESR-01, 02, 03, 05, 06, 07, 09, 12, 13, 15, 17–22, 25, 27, 31, 32, 35, 36 and 37); (ii) Intermediate (ESR-08, 11, 14, 16, 23, 26, 28, 29, 34, 38, 40, 42, 43, 46, 48, 50, 53, 55–58, and 64); and (iii) Slow growing (ESR-04, 10, 24, 30, 33, 39, 41, 44, 47, 49, 51, 52, 54, 59, 60–63, and 65). Although most of the *S. sclerotiorum* isolates has attained full mycelial growth and filled up the 90 mm Petri plates within 4–5 days whereas other isolates namely ESR-04, 10, 24, 26, 28, 30, 49, 52, and 62 were taken 7–9 days for attaining the full growth and in filling the 90 mm Petri plate with the mycelial mat. Among these *S*. *sclerotiorum* isolates collection, most of them were had whitish mycelial growth with a smooth texture but the remaining isolates ESR-02, 06, 08, 12, 14, 17, 21, 31, 42, and 48 had an off-white color.

All the distinct *Sclerotinia* isolates were categorized based on the number and rate of sclerotia produced by them at a given time point into three groups: (i) High producer (ESR-20, 23, 26, 39, 46, 51, 58, and 65), (ii) Intermediate (ESR-01–19, 21, 22, 24, 25, 27–29, 31–38, 40–45, 47–49, 52–57, 59–64), and (iii) Low producer (ESR-30 and 50). In the majority of the isolates, sclerotia were observed produced within 6–9 days after growth. The sclerotia of isolates ESR-26, 27, 44, 49, 55 and 65 were larger in size (2.4–2.5 mm in diameter), while those of ESR-01, 02, 04, 09, 13, 18 were smallest (1.8–1.9 mm in diameter). The isolates varied in their sclerotial length (2.9–6.6 mm), however, the sclerotia of isolate ESR-65 had a maximum length (6.6 mm) (Table S1).

**Figure 2:**
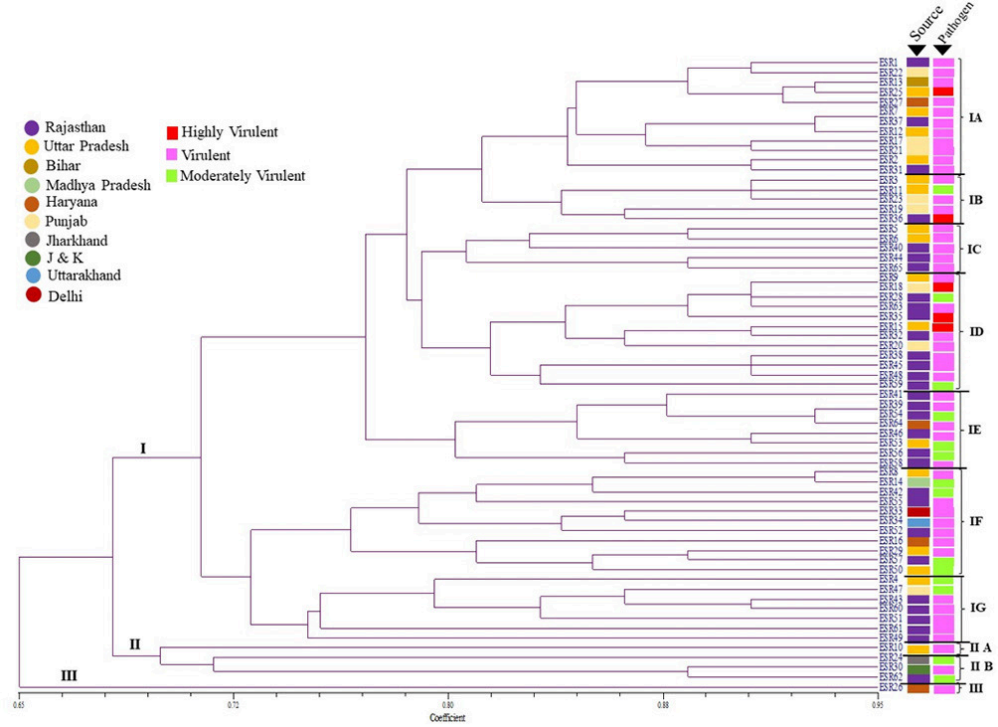
Dendrogram derived from the morphological characters of 65 different *S*. *sclerotiorum* isolates, based on growth, color, texture, shape and size of the mycelia and sclerotia grown on PDA medium and their respective pathogenicity over *Brassica* species.

The morphological features among *S. sclerotiorum* collections from *Brassica* field of different agronomic regions in India (Figure 1), were used in genetic diversity analysis. The dendrogram generated (Jaccard's coefficient) have three distinct clusters at 65% of distance (Figure 2). Cluster I, with the highest degree of genetic variation, comprises 60 isolates from different regions; cluster II has four isolates, collected from distinct geographical locations or provinces, and cluster III contains one isolate, from Haryana. The robustness of the tree was estimated by bootstrap resampling, and the bootstrap values for clusters I, II, and III were very high, 100, 99, and 100%, respectively. Out of three clusters, cluster I was the largest and differentiated into seven distinct sub-clusters: IA-IG. Almost 99% of the isolates from different provinces fell under these sub-clusters. The majority of the *S. sclerotiorum* isolates from Rajasthan were grouped in the sub-cluster IC to IG (83%) and rest were distributed across the dendrogram. Cluster II had 4 isolates were grouped into two sub-clusters IIA and IIB. All the four isolates one in IIA and three in IIB were from four different states whereas single isolate present in cluster III was from Haryana region. Individual isolates from Jharkhand and J&K distributed in sub-cluster IIB. Interestingly out of 4 isolates in cluster II, 2 of the isolates one each from Rajasthan and Jharkhand was moderately virulent. One notable thing in the dendrogram was the distribution of the highly virulent isolates, one from each states UP, Punjab and Rajasthan had clustered into sub-clad ID whereas another highly virulent isolates one each from UP and Rajasthan falls under IA and IB, respectively.

### Pathogenicity

Plants inoculated with *S. sclerotiorum* developed necrotic and bleached lesions 6–9 days after inoculation, and white cottony mycelial growth appeared on stem surface after successful infestation. Pathogenic variability test conducted on 4 *Brassica* differentials has shown that all the isolates collected were pathogenic and able to develop stem rot disease after infection. However, the aggressiveness of the isolates was observed varies contrarily on all the host differentials and thus diverse groups were identified for each character by comparing the pathologically quantitative characteristics i.e., stem lesion length and percentage of disease incidence on different host differentials. All the 65 *S. sclerotiorum* isolates from different geographical regions showed variations in pathogenicity in respect of stem lesion length. After artificial inoculation of *S. sclerotiorum*, the typical water-soaked lesion was observed on the stems of host differentials. On the basis of longest and shortest stem lesion formed by different geographical isolates of four *Brassica* species, following 4 groups were made: Group I: (*Brassica juncea* var. NRCDR-02), Group II: (*B. rapa* var. toria), Group III: (*B. rapa* var. yellow sarson), and Group IV: (*B. rapa* var. brown sarson). Group I, composed of 9 isolates viz., ESR−12, 15, 17–19, 25, 35, 36, 65 produced longer stem lesion and among them ESR-36 produced the longest lesion i.e., 49.0 cm, while ESR-26, 40 and 64 isolates produced shortest stem lesion i.e., 1.0 cm (range:1.0–49.0 cm). Similarly, other 6 groups also showed pathogenic variability. Based on the stem lesion formation, out of 7 groups, ESR-36 and ESR-60 isolates were found producing longest and shortest stem lesion on the inoculated stem, respectively.

The results of pathogenicity tests conducted by using the 65 isolates of *S. sclerotiorum* over the 4 different *Brassica* differential has shown that the isolates had differences in their pathogenicity, infectivity and disease severity on tested *Brassica* cultivars and are presented in Table 1. The disease severity was measured in terms of Mean Disease Severity (MDS) by measuring the lesion length appeared on infected stem and that provides the basis for recording the virulence of individual isolate as low (MDS: < 3 cm), medium (MDS: < 3–10 cm) and high (MDS: > 10 cm). Thus, based on the disease severity index of the isolates *S. sclerotiorum* collection was distributed into three groups viz., highly virulent (5 isolates), virulent (46 isolates), and moderately virulent (14 isolates) based on the variations observed by analyzing the mean disease severity index in the treated mustard variety (Table 5). The un-inoculated and control plants of test variety inoculated with plain agar media showed no symptoms. The *Sclerotinia* isolates were further re-isolated and cultured from the infected *Brassica* plants following Koch's postulates.

**Table 5:** Virulence grade for *Sclerotinia sclerotiorum* population assigned based on disease severity assessment and GenBank accession numbers for the ITS sequences.

**Strain No**.	**Source (Host)**	**Province**	**Pathogenicity of the isolate**	**GenBank accession no**.
				
ESR 1	*B. juncea*	Rajasthan	Virulent	MF408233
ESR 2	*B. juncea*	UP	Virulent	MF408234
ESR 3	*B. rapa*	UP	Virulent	MF408235
ESR 4	*B. juncea*	UP	Moderately Virulent	MF408236
ESR 5	*B. rapa*	UP	Virulent	MF408237
ESR 6	*B. juncea*	UP	Virulent	MF408238
ESR 7	*B. juncea*	UP	Virulent	MF408239
ESR 8	*B. juncea*	UP	Virulent	MF408240
ESR 9	*B. juncea*	UP	Virulent	MF408241
ESR 10	*B. juncea*	UP	Virulent	MF408242
ESR 11	*B. juncea*	UP	Moderately Virulent	MF408243
ESR 12	*B. juncea*	UP	Virulent	MF408244
ESR 13	*B. rapa*	Bihar	Virulent	MF408245
ESR 14	*B. juncea*	MP	Moderately Virulent	MF408246
ESR 15	*B. juncea*	UP	Highly Virulent	MF408247
ESR 16	*B. juncea*	Haryana	Virulent	MF408248
ESR 17	*B. juncea*	Punjab	Virulent	MF408249
ESR 18	*B. juncea*	Punjab	Highly Virulent	MF408250
ESR 19	*B. juncea*	Punjab	Virulent	MF408251
ESR 20	*B. juncea*	Punjab	Virulent	MF408252
ESR 21	*B. juncea*	Punjab	Virulent	MF408253
ESR 22	*B. juncea*	Punjab	Virulent	MF408254
ESR 23	*B. juncea*	Punjab	Virulent	MF408255
ESR 24	*B. juncea*	Jharkhand	Moderately Virulent	MF408256
ESR 25	*B. juncea*	UP	Highly Virulent	MF408257
ESR 26	*B. juncea*	Haryana	Virulent	MF408258
ESR 27	*B. juncea*	Haryana	Virulent	MF408259
ESR 28	*B. juncea*	Rajasthan	Moderately Virulent	MF408260
ESR 29	*B. juncea*	UP	Virulent	MF408261
ESR 30	*B. juncea*	J & K	Virulent	MF408262
ESR 31	*B. juncea*	Rajasthan	Virulent	MF408263
ESR 32	*B. juncea*	Rajasthan	Virulent	MF408264
ESR 33	*B. juncea*	Delhi	Virulent	MF408265
ESR 34	*B. juncea*	Uttarakhand	Virulent	MF408266
ESR 35	*B. juncea*	Rajasthan	Highly Virulent	MF408267
ESR 36	*B. juncea*	Rajasthan	Highly Virulent	MF408268
ESR 37	*B. juncea*	Rajasthan	Virulent	MF408269
ESR 38	*B. juncea*	Rajasthan	Virulent	MF408270
ESR 39	*B. juncea*	Rajasthan	Virulent	MF408271
ESR 40	*B. juncea*	Rajasthan	Virulent	MF408272
ESR 41	*B. juncea*	Rajasthan	Virulent	MF408273
ESR 42	*B. juncea*	Rajasthan	Moderately Virulent	MF408274
ESR 43	*B. juncea*	Rajasthan	Virulent	MF408275
ESR 44	*B. juncea*	Rajasthan	Virulent	MF408276
ESR 45	*B. juncea*	Rajasthan	Virulent	MF408277
ESR 46	*B. juncea*	Rajasthan	Virulent	MF408278
ESR 47	*B. juncea*	Punjab	Moderately Virulent	MF408279
ESR 48	*B. juncea*	Rajasthan	Virulent	MF408280
ESR 49	*B. juncea*	Rajasthan	Virulent	MF408281
ESR 50	*B. juncea*	UP	Moderately Virulent	MF408282
ESR 51	*B. juncea*	Rajasthan	Virulent	MF408283
ESR 52	*B. juncea*	Rajasthan	Virulent	MF408284
ESR 53	*B. juncea*	UP	Moderately Virulent	MF408285
ESR 54	*B. juncea*	Rajasthan	Moderately Virulent	MF408286
ESR 55	*B. juncea*	Rajasthan	Virulent	MF408287
ESR 56	*B. juncea*	Rajasthan	Moderately Virulent	MF408288
ESR 57	*B. juncea*	Rajasthan	Moderately Virulent	MF408289
ESR 58	*B. juncea*	Rajasthan	Virulent	MF408290
ESR 59	*B. juncea*	Rajasthan	Moderately Virulent	MF408291
ESR 60	*B. juncea*	Rajasthan	Virulent	MF408292
ESR 61	*B. juncea*	Rajasthan	Virulent	MF408293
ESR 62	*B. juncea*	Rajasthan	Moderately Virulent	MF408294
ESR 63	*B. juncea*	Rajasthan	Virulent	MF408295
ESR 64	*B. juncea*	Haryana	Virulent	MF408296
ESR 65	*B. juncea*	Rajasthan	Virulent	MF408297

*GenBank accession IDs of each isolate based on ITS sequence information*.

### SSR genotyping

**Figure 3:**
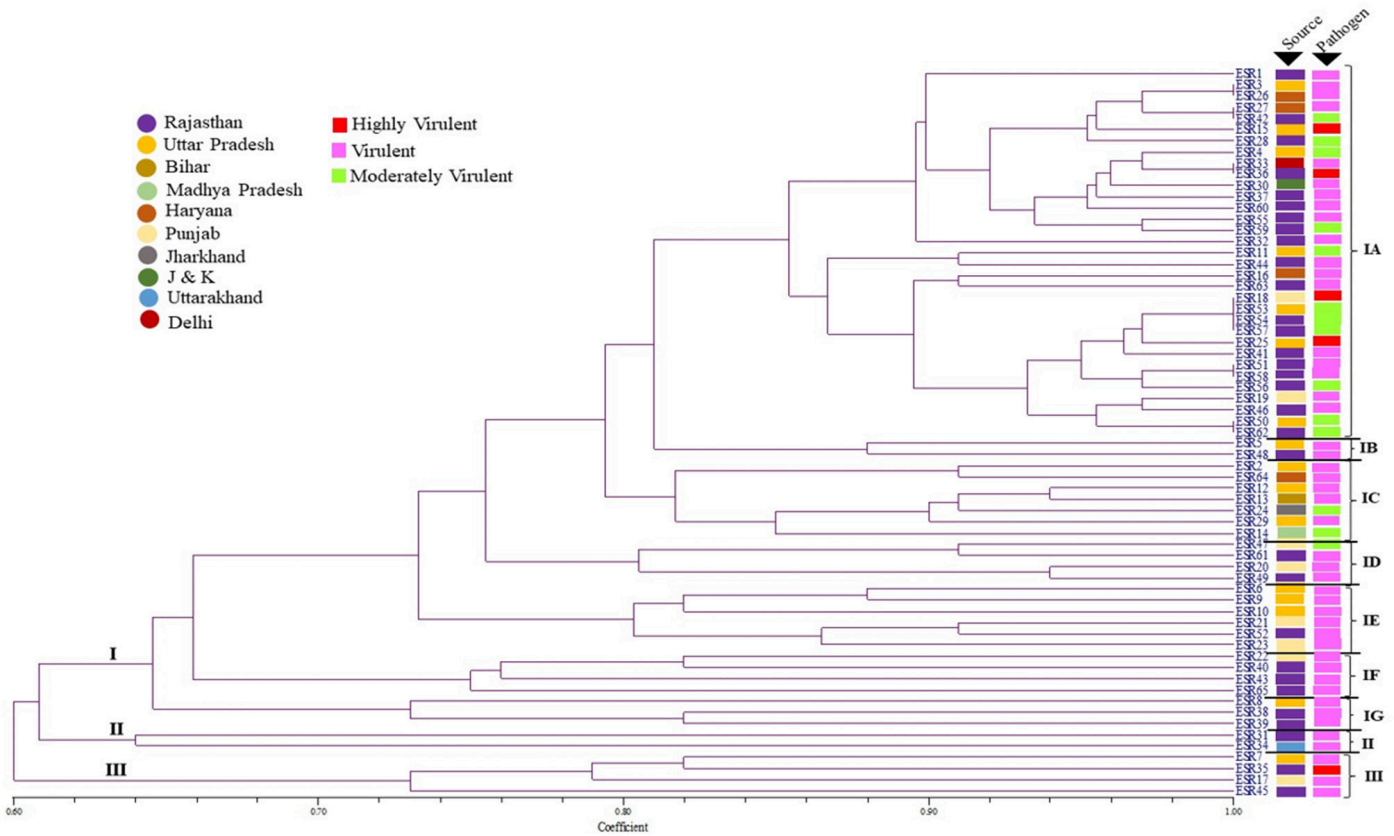
Un-weighted pair group method with arithmetic mean (UPGMA) dendrogram showing the genetic relationships between the 65 isolates of *S. sclerotiorum* assessed in this study, based on simple sequence repeat (SSR) markers (Jaccard's coefficient) and their respective pathogenicity over the *Brassica* species.

The population dynamics study of the pathogen plays a vital role in deciphering the mechanism behind behavioral pattern as well as in understanding the distribution pattern of the pathogen in distinct geographical areas where host species envisioned frequently by the infective pathogen. In this direction, the genetic diversity of *S. sclerotiorum* isolates were analyzed by simple sequence repeat (SSR) based fingerprinting. All the 25 SSR primers were analyzed after optimizing the melting temperature (Tm) for polymorphism assay on collective set of 65 isolates comprising representatives from the major *Brassica* growing states of India included in the study: Rajasthan (31), Uttar Pradesh (16), Punjab (8), Haryana (4), Delhi (1), Bihar (1), Madhya Pradesh (1), Uttarakhand (1), Jharkhand (1), and Jammu & Kashmir (1). Among the genome-wide distributed primers, only four primers (9I, 12L, 14N, and 17Q) has generated multiple bands whereas remaining primers were produced a single polymorphic band (Table 3). Two primers 9I and 17Q were observed produced 2 polymorphic bands of 100–500 bp size range per isolate. Subsequently other two primers 12L and 14N have produced 4 polymorphic bands within a range of 200–1,000 bp (Figures S1–S9). The differences in genetic diversity among the *S. sclerotiorum* pathogen population obtained through SSR fingerprinting in this study were found reproducible. Subsequently, UPGMA dendrogram drawn based on presence and absence of the banding patterns showed substantial diversity among the isolates. Based on the topology and similarity indices of the dendrogram, the isolates were grouped into three major clades and each clade was signified by a roman numeral (group I to III; Figure 3).

The detailed analysis of the dendrogram revealed, maximum numbers of isolates grouped in clade I and it was the largest and based on the similarity indices the clade I was further separated into seven divergent sub-clades: IA-IG. Among these sub-clades, IA, IF and IG harbors nearly 60% of the *S. sclerotiorum* isolates from Rajasthan whereas, remaining 40% were distributed in other clades of the dendrogram. The other two major clades, clade II and III had 2 and 4 isolates and in which 1 isolate in clade II and 2 isolates in clade III were from Rajasthan. Almost all the isolates from UP were clustered in clade I under sub-clades IA, IB, IC, IE, and IG. But, isolates from Punjab has a different trend of distribution across the dendrogram. All the isolates from Haryana were clustered in sub-clades IA and IC. Individual isolates from Delhi and J&K distributed in sub-clade IA, from Bihar, Jharkhand, and MP distributed in IC, and one isolate from Uttarakhand distributed in the major Clade II. The distribution of highly virulent isolates in dendrogram was found interesting were two from UP and one isolates from each Rajasthan and Punjab had clustered into IA sub-clade whereas another highly virulent isolate from Rajasthan was clustered in clade III. The prominent genetic diversity feature in clustering was observed in sub-clade IE, IF, IG and clade II, III those included exclusively virulent or highly virulent isolates and more interestingly these clades had at least one isolate from individual state included in this study.

### ITS sequence analysis

The fungal-specific ITS4-ITS5 universal primers pair was used in amplifying the internal transcribed spacer (ITS) region from the DNA of all the 65 *S. sclerotiorum* isolates collected from infected *Brassica* crop grown in the diverse genetic background. The amplicon lengths and purity was estimated by gel electrophoresis and found about 600 bp in size (Figure S11). The sequence analysis result of the rRNA data by NCBI BLAST tool observed connoted the morphological variability and supported their individual existence. Further, in NCBI GenBank database the 99–100% closest match was found with *S. sclerotiorum*. The sequence data information for the ITS rDNA region of all the 65 *S. sclerotiorum* isolates obtained in this study were submitted in the GenBank database of the NCBI (GenBank Accession numbers MF408233-MF408297; Table 5).

**Figure 4:**
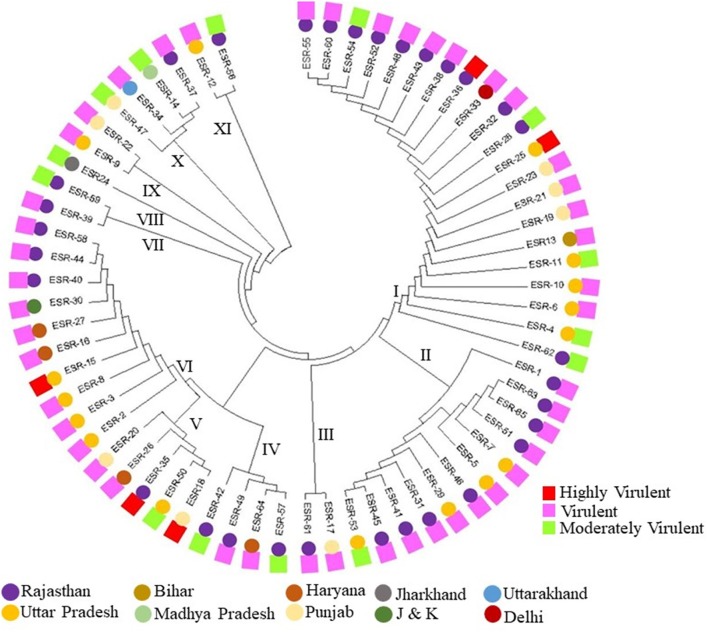
Phylogenetic analysis of *S. sclerotiorum* isolates based on rONA ITS sequences by Neighbor-Joining method and respective pathogenicity over *Brassica* species.

Based on the ITS sequence information a dendrogram was constructed by using the ClustalW and Mega7.0 software with the Neighbor-Joining method (Figure 4). The resulted phylogenetic tree was clustered in total 11 clades and six of them were formed major clades, four clades with two members each and one clade with a single member that clearly indicates the distribution of the isolates across dendrogram irrespective of their properties of pathogenicity or geographic origin. Isolates from Rajasthan and UP were found distributed in nine and six different clades, respectively, whereas isolates from Punjab were found distributed in five different clades. Clade I was found to be the largest and consisted of 21 divergent isolates from Rajasthan, UP, Punjab, Bihar, and Delhi with varied pathogenicity (highly virulent, virulent and moderately virulent). Clade II had virulent and moderately virulent isolates from Rajasthan and UP whereas isolates from Haryana were all virulent and distributed in clades IV, V and VI. Clade VII had only two isolates and both of them were from Rajasthan and virulent. Interestingly, the clade IV and V were also found heterogeneous as of clade I in terms of the isolates of different region and their virulence features. The isolate from Jharkhand was found distinctly in the clade VIII and was moderately virulent. Remarkably clade X had four isolates from the four different states. Two pathogenic isolate each from Rajasthan and Punjab were formed clade III, from UP and Punjab have formed clade IX and from UP and Rajasthan were formed clade X. One moderately pathogenic isolate from Jharkhand formed the single member clade VIII. The highly pathogenic isolates from UP, Rajasthan, and Punjab were distributed in Clades I, V, and VI.

The ITS sequence analysis of all the isolates in the study depicted on an average 95–100% of sequence similarity that entails conspecific nature of the isolates and their host from which the isolates were collected irrespective of different geographic regions.

## Discussion

The incidence of stem rot disease in India, especially in oilseed *Brassica*, has been known to damage the crops since from the mid-1990 but the genetic diversity of the pathogen especially in oilseed *Brassica* has not been comprehensively studied so far which is primarily required for understanding the mechanism of the pathogenesis and virulence of the pathogen. From the last decade, the stem rot disease in *Brassica* poses a serious threat to the crop every year in India and causes significant damage to the crop in terms of yield and economic losses to the farmers due to lack of effective management practices and other control measures. From the frequent disease occurring reports and extent of its severity over the oilseed crops, this pathogen has drawn attention and interest of the researchers to analyze the host-pathogen interaction in a broader spectrum as the mustard growing farmers experiencing this problem almost every years during the cropping season. The present investigation thoroughly reporting the existence of *S. sclerotiorum* pathogen in mustard fields and the genetic diversity among the population distributed throughout the *Brassica* growing regions of India.

The field-based crop-specific disease survey in different states of the India has shown the high occurrence and well-dispersed existence of the stem rot disease that commonly associated with the scorched lesion on the stem of the infected plants and sclerotia infested soil samples in the severely affected crop field. The regular appearance of the stem rot disease in *Brassica* indicates it is a recurrent menace for the crop and the lack of promising resistance source to the pathogen in existing cultivated varieties. Although the isolates in the present investigation were collected from *Brassica* field only but even though they were found genetically diverse in terms of their culture conditions and in morphological appearance as well. This might be due to the divergent geographical location from where the isolates have been collected. The evaluation of the pathogenicity of different *Brassica* cultivars has revealed that there was differential susceptibility in host species against different isolates of the *S. sclerotiorum* and thus based on the pathogenicity assay and virulence properties the pathogen population was grouped into three subgroups highly virulent, virulent and moderately virulent isolates. Depending on their virulence all the isolates resulted in the development of lesions of white patches of a different length over the infected stem of the *Brassica* cultivars that indicates the existence of genetically diverse and variable population of *S. sclerotiorum*. The pathogenicity assay and virulence variability test have shown that out of 65 *S. sclerotiorum* isolates 5 of them were highly virulent, 46 were virulent and 14 isolates were falls under moderately virulent category and they disseminated through either contaminated soil or seed across *Brassica* growing regions in India. The genetic diversity analysis by all the means followed in this study has clearly shown there were not many differences exists in terms of virulence and in groups that comprised from the isolates of different virulence nature belongs to different localities. Our results are in accordance with the observations made in previous studies where aggressiveness of the isolates are being independent of their geographic distribution (Atallah et al., 2004; Xie et al., 2014). Hence, the nature of the virulence of the *S. sclerotiorum* pathogen possesses a potential threat to the planting of *Brassica* cultivars susceptible to the stem rot disease.

*Sclerotinia sclerotiorum* apart from reproducing asexually by myceliogenic germination of sclerotia it is also able to produce sexually through apothecia and ascospores formation. The diversity analysis and genetic architecture of *S. sclerotiorum* have shown the existence of homogeneity across the isolates with little variation in 18 and 28 s rDNA regions (Kohli et al., 1995). The genetic diversity of the pathogen, severity of the disease and frequency of its occurrence are the important factors that directly correlates with the epidemics of the disease at the regional level and hence their comprehensive studies are the foremost requirement for understanding the behavioral pattern of the pathogens for effectively design the management practices for the control of this disease more efficiently. The polymorphism assay with the genome-wide distributed SSR markers has been proven effective in studying the existence of genetic diversity within the pathogen population. The SSR is the co-dominant molecular markers and it is a PCR-based robust technique that amplifies the putative repeat regions of the DNA and therefore it is able to identify a higher level of polymorphism than PCR-based another less robust markers like RAPD and hence it has been extensively used for analyzing the fungal population. The genetic diversity among *S. sclerotiorum* isolates based on the morphological features and SSR fingerprinting had observed quite diverse and reveals that some of the isolates grouped together belonging to the same region while others from the same region distributed across the dendrogram. The morphological features of the isolates and their SSR fingerprinting confirms the existence of a highly variable population of *S. sclerotiorum* even within the same crop and the region. However, among some isolates of *S. sclerotiorum* those belonging to the same geographic regions has been found having lower genetic diversity and that might be due to the locally adapted cropping system where introduction of newer varieties are usually not preferred over the existing one and hence diversity of the pathogen remained to stagnate due to lack of introduction of the newer genotypes of the pathogen in the area under cultivation.

The result of the present investigation depicting the genetic diversity of *S. sclerotiorum* population based on the morphological parameters and SSR analysis has been observed in coherence with earlier findings of the studies made on the same pathogen but in different host species. In eggplant, the genetic diversity analysis of *S. sclerotiorum* population by using the SSR and RAPD molecular markers, Tok et al. (2016) had shown the existence of high level of heterogeneity in the pathogen populations and presence of different isolates of *S. sclerotiorum* pathogen in the same regions. The presence of high heterogeneity in genetic composition of the *S. sclerotiorum* isolates in different regions of Turkey has reflected by their distribution across the dendrogram in different groups made from the RAPD and SSR profiling. In a similar study, Barari et al. (2013) have characterized the genetic diversity within the population of *S. sclerotiorum* isolates from Iran and the variations among them were projected by SSR profiling. The depiction of variability in closely related isolates by SSR markers demonstrated its effectiveness in analyzing the genetic diversity among *S. sclerotiorum* pathogen. In chickpea, the use of RAPD, ITS-RFLP, ITS sequencing, and mycelial compatibility groupings (MCG) has shown the limited variability among the distinct *S. sclerotiorum* isolates possess the higher genetic homogeneity and partially correlated with their geographical origin (Mandal and Dubey, 2012). The robustness and reproducibility features of the SSR markers make it more effective and reliable marker for deciphering the genetic relationship among population studies and thus the phylogenetic analysis of *S. sclerotiorum* based on the SSR genotyping revealed the existence of wider genetic diversity among its population. The present findings are in connotation with the earlier reports that molecular typing by SSR is an efficient technique for establishing the genetic relationship among the diverse population and it is being preferred over other molecular tools for diversity analysis.

Further, the ITS (internal transcribed spacer) sequence based molecular phylogeny was established by using the ITS region as a genetic marker to investigate the genetic association within the *S. sclerotiorum* isolates. This phylogenetic study has facilitated the revelation of the evolutionary relationship within *S. sclerotiorum* species collected from a single host *Brassica* species from different geographical regions of India. Based on the ITS phylogeny all the 65 isolates investigated in the present study were observed grouped into six major and five minor evolutionary lineages of *S. sclerotiorum* isolates collected from different mustard growing states of India. In all the lineages deviation in virulence was not so evident and this suggests that the isolates from single host species may not differ ominously in their pathogenicity. The least variation in virulence of the pathogen has been observed in the case where isolates from defined geographical regions were taken into the investigation (Alvarez and Molina, 2000; Atallah et al., 2004; Sexton and Howlett, 2004). The differences in virulence can be more evident and conclusive when isolates from the widely distant geographical area will be taken for comparison. Kull et al. (2004) has found the conclusive evidence for the host specialization among *S. sclerotiorum* isolates.

Although, there is no report of any toxin is usually produced by the *S. sclerotiorum* pathogen it has been observed the production of oxalic acid (Cessna et al., 2000) and extracellular lytic enzymes production (Riou et al., 1991) invariably by all the isolates during pathogenesis. However, extensive biochemical and physiological studies are required to investigate the production of any natural mycotoxins by the pathogen if any like other fungus and further needs to find the effect of those toxins on the host plants and in herbivores consuming those plants. In this study, we attempted to verify the authenticity of *S. sclerotiorum* isolates by using the *Sclerotinia*-specific PCR primers and found all the geographical isolates (65) investigated were genetically diverse.

Development of the Genetic fingerprinting database for distinguishing the pathogen diversity between and within the fungal species is one of the foremost requirement for determining the origin and evaluation of the pathogen. Unequivocally, environmental factors are also influencing the properties of the pathogen and play crucial roles in determining the genetic differentiation within the pathogen population (Weller, 1988). The existence of a mixed population of *S. sclerotiorum* within a region could be due to the possible human interventions like the movement of infested soil through seeds, packaging materials or agricultural equipment that might carry the pathogen from one region to another. Results of the present study, as well as evidence from the previous studies, indicates the minute changes in physiological properties of the pathogen like changes in virulence due to evolutionary changes happening in due course of time that brings changes among genetically distinct populations of *S. sclerotiorum* isolates. Such changes may happen because of the varying force of selection over pathogen imposed by the selected set of genes of the host germplasm being tolerant to the invasive pathogen. The genes involved in host defense system and coding for the virulence factors in pathogen have been observed comparatively much more vulnerable for accumulating the mutation under positive selection pressure than the genes those regulating the basic physiological processes in the organism (Matute et al., 2008). As a consequence, in most of the host-pathogen interaction pathogen often adapts themselves to the evolutionary changes arises in response to the variation in the host genotype.

## Conclusion

The current investigation reports the studies on morphological features, pathogenicity variance over the *Brassica* cultivars, and genetic diversity based on molecular characteristics of the *S. sclerotiorum* isolates population collected from 65 locations of the 10 major *Brassica* growing states of India. This is the first comprehensively studied report on *S. sclerotiorum* isolates especially from *Brassica* field of India that indicates the existence of broader genetic variations among the pathogen and this would facilitate the plant breeders to use the information in *Brassica* breeding program for stem rot disease resistance development. The SSR-based markers utilized in present investigation for diversity analysis can be effectively used for identification and analysis of *S. sclerotiorum* population from various host species. The present study offers the basis for increasing the basic understanding of host-pathogen interaction and also for deciphering the genes and associated pathways involved in combating the pathogen invasion. The evidence of the present study may provide a broader understanding of the pathogen virulence and that will facilitate the development of the effective disease management strategies based on molecular breeding and other advanced approaches.

## Author contributions

PS, NG, and LP conceived and obtained funding from ICAR-EMR project for Sclerotinia work in Indian mustard. PS and VS collected isolates from diseased plants and along with LP done the morphological characterization and pathogenicity evaluation. AS and MR obtained isolates from PS, carried out microsatellite genotyping. NG and RB do the ITS sequencing of *S. sclerotiorum* isolates and wrote the paper. NG and DM carried out clustering and phylogenetic analyses of microsatellite data and ITS sequences. RB and VS edited the manuscript.

### Conflict of interest statement

The authors declare that the research was conducted in the absence of any commercial or financial relationships that could be construed as a potential conflict of interest.
