# Supplementary figures and images for: Genetic Diversity Studies Based on Morphological Variability, Pathogenicity and Molecular Phylogeny of the Sclerotinia sclerotiorum Population From Indian Mustard (Brassica juncea)
Source: Front Microbiol. 2018 Jun 5;9:1169. doi: 10.3389/fmicb.2018.01169 (PMC5996862; doi:10.3389/fmicb.2018.01169)

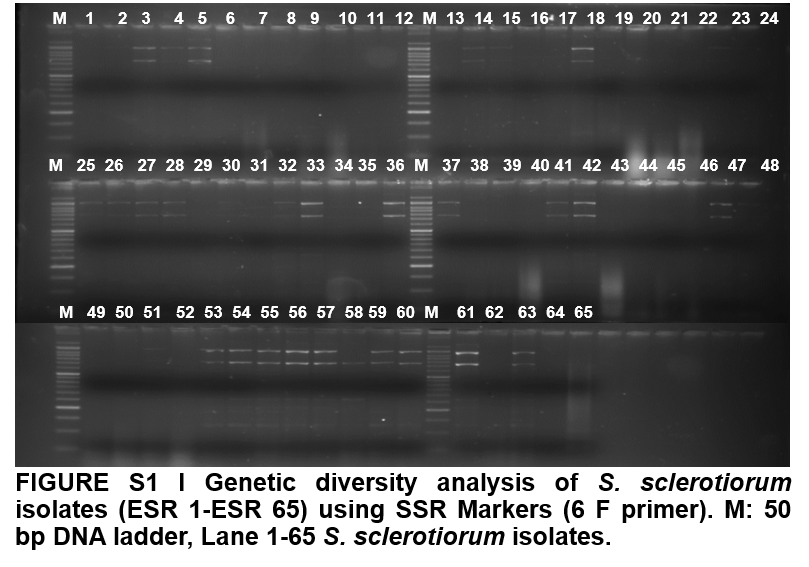

Supplement: Supplementary file 1 [file Image_1.TIF]

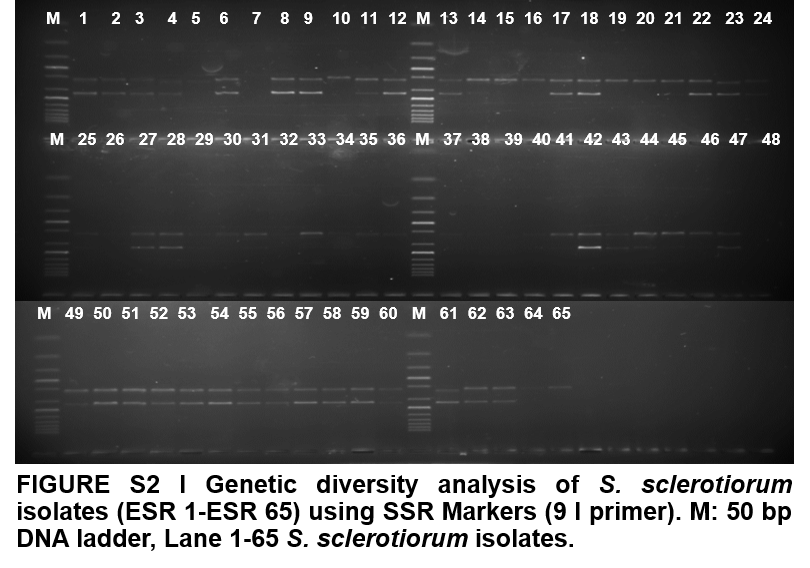

Supplement: Supplementary file 2 [file Image_2.TIF]

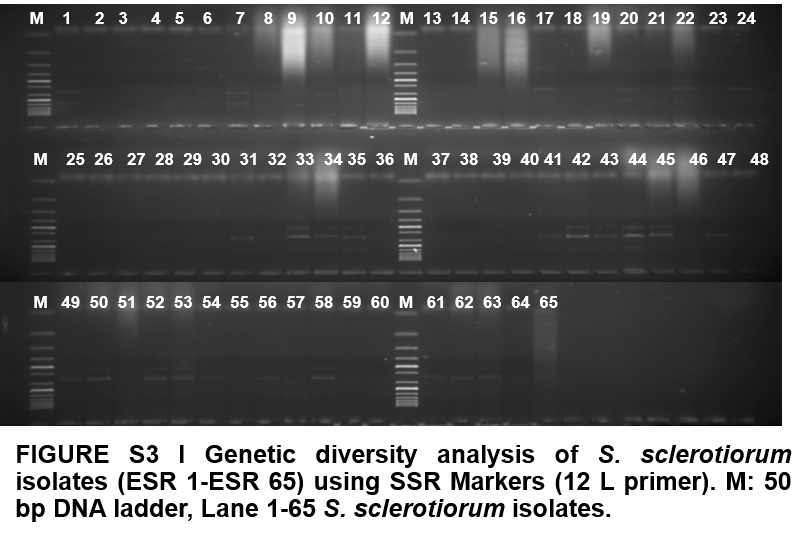

Supplement: Supplementary file 3 [file Image_3.TIF]

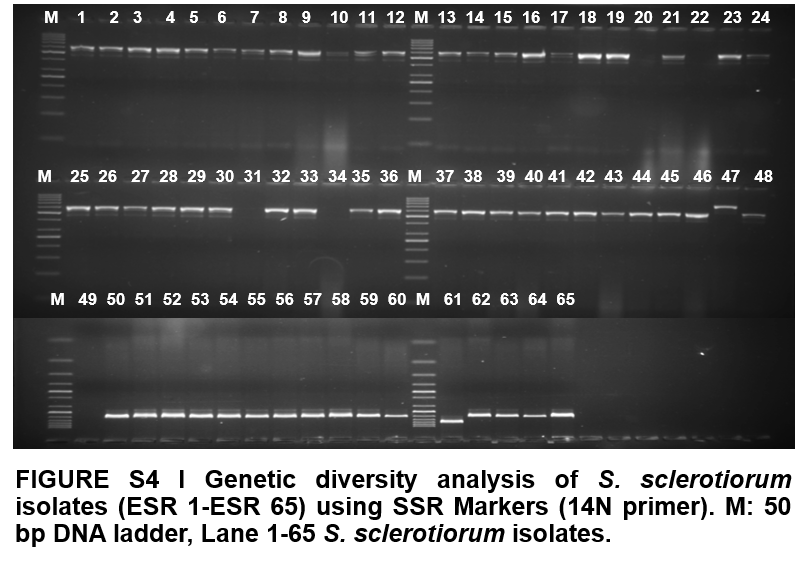

Supplement: Supplementary file 4 [file Image_4.TIF]

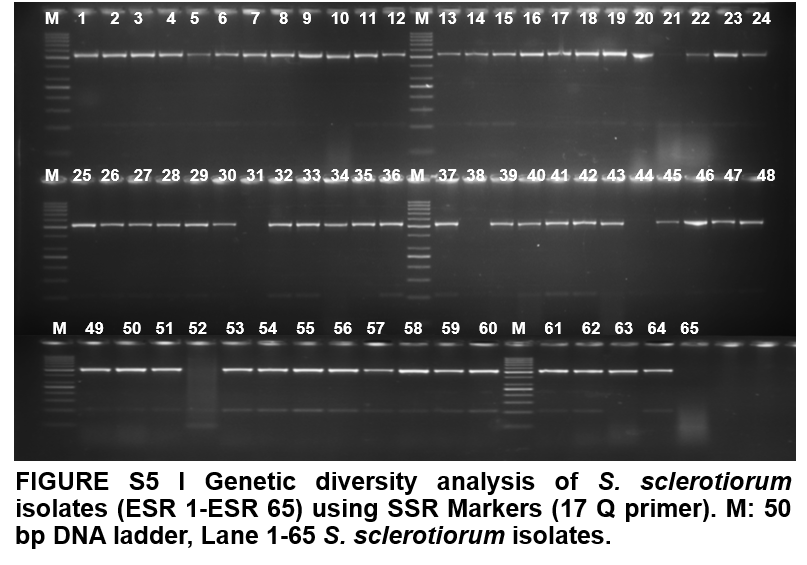

Supplement: Supplementary file 5 [file Image_5.TIF]

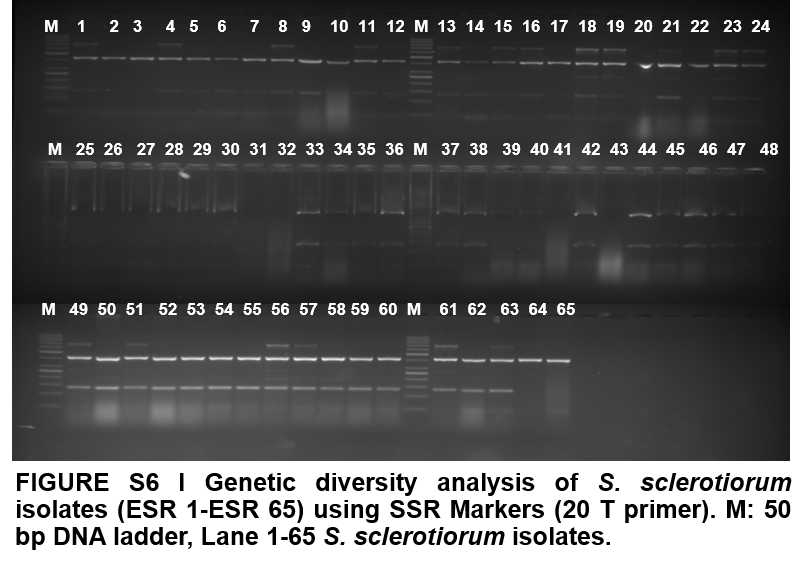

Supplement: Supplementary file 6 [file Image_6.TIF]

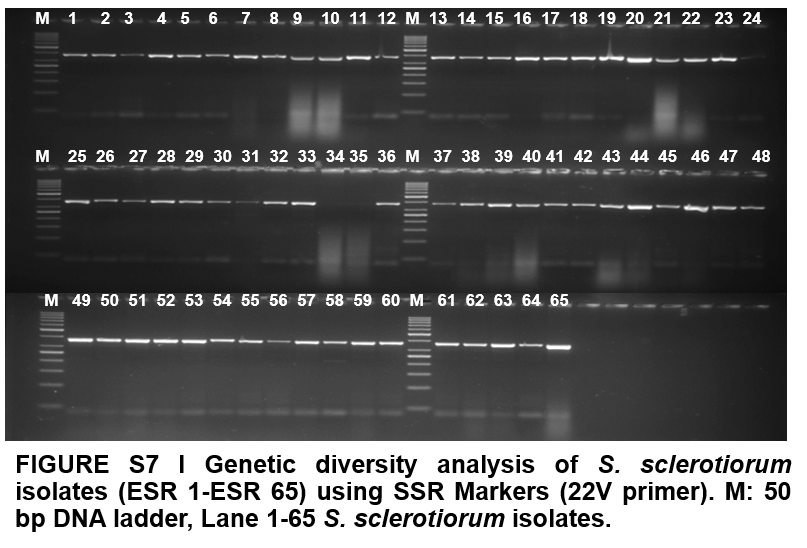

Supplement: Supplementary file 7 [file Image_7.TIF]

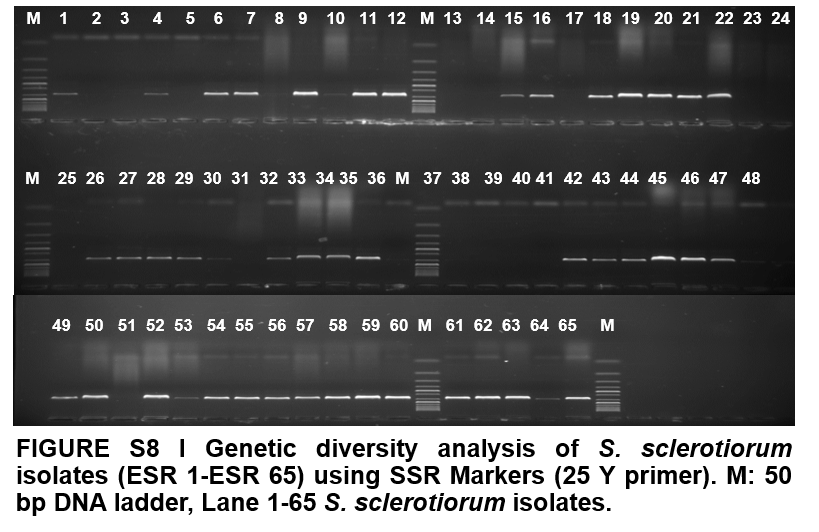

Supplement: Supplementary file 8 [file Image_8.TIF]

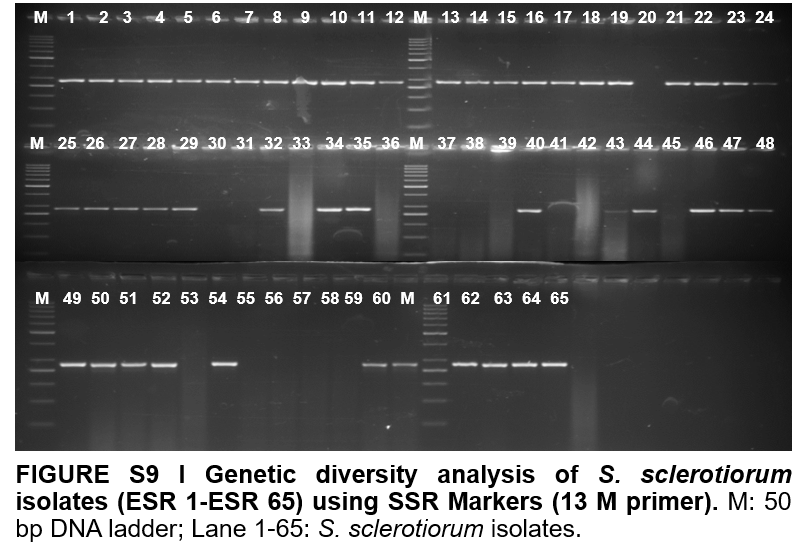

Supplement: Supplementary file 9 [file Image_9.TIF]

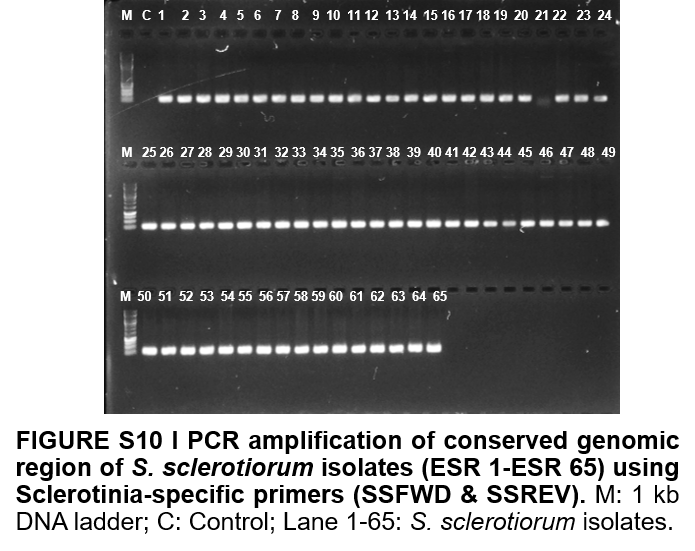

Supplement: Supplementary file 10 [file Image_10.TIF]

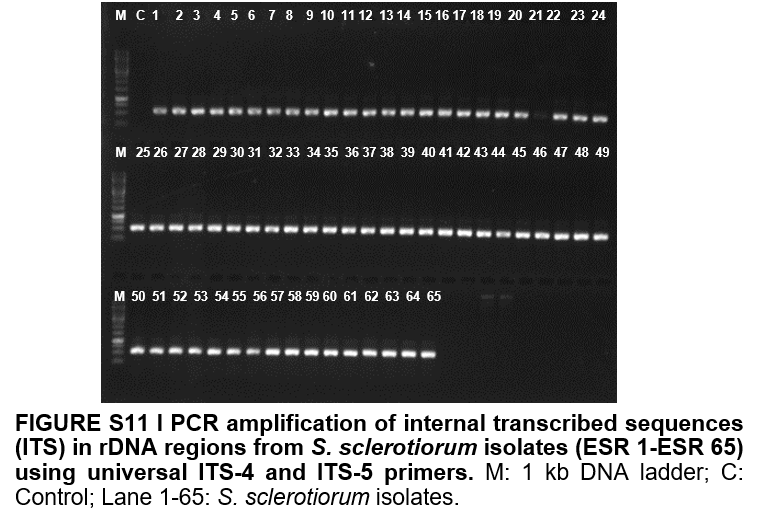

Supplement: Supplementary file 11 [file Image_11.TIF]
